# Determinants of early postnatal care attendance: analysis of the 2016 Uganda demographic and health survey

**DOI:** 10.1186/s12884-020-02866-3

**Published:** 2020-03-16

**Authors:** Patricia Ndugga, Noor Kassim Namiyonga, Deogratious Sebuwufu

**Affiliations:** 1 Department of Population Studies, School of Statistics and Planning, Makerere University, Kampala, Uganda; 2 Uganda Bureau of Statistics, Kampala, Uganda; 3 Uganda Ministry of Health, Kampala, Uganda

**Keywords:** Early postnatal care, Uganda, Demographic and health survey, Place of delivery

## Abstract

**Background:**

The first 2 days after childbirth present the highest risk of dying for a mother. Providing postnatal care within the first 2 days after childbirth can help avert maternal mortality because it allows early detection of problems that could result in adverse maternal health outcomes. Unfortunately, knowledge of the uptake of early postnatal care (EPNC), which is imperative for informing policies aimed at reducing maternal mortality, remains low in Uganda. Therefore, the purpose of this study is to investigate the determinants of early postnatal care attendance among Ugandan women.

**Methods:**

This study was based on nationally representative data from the 2016 Uganda Demographic and Health Survey. The study sample comprised 5471 women (age 15–49) who delivered a child in the 2 years preceding the survey. We used logistic regression to identify factors associated with use of early postnatal care.

**Results:**

Our findings showed that 50% of mothers used EPNC services for their most recent delivery in the 2 years preceding the survey. Women’s residence, education level, religion, wealth status, marital status, occupation, antenatal care attendance, place of delivery, birth order, perceived accessibility of health facilities, and access to mass media messages were associated with greater use of EPNC. The percentage of women receiving EPNC was much higher among women who delivered at a health facility, either a public facility (63%) or private facility (65%), versus only 9% among women who delivered at home. Multivariate analysis showed that delivery at a health facility was the most important determinant of early postnatal care attendance.

**Conclusions:**

To increase mothers’ use of EPNC services and improve maternal survival in Uganda, programs could promote and strengthen health facility delivery and ensure that EPNC services are provided to all women before discharge. Even so, the fact that only about two-thirds of women who delivered at a health facility received early postpartum care shows substantial room for improvement. Interventions should target women who deliver at home, women who attend fewer than four antenatal care visits, and women with a primary education.

## Background

Globally, an average of 830 women die every day from preventable causes related to pregnancy and childbirth; of these deaths, almost all (99%) occur in developing countries [1, 2]. More than 60% of global maternal deaths occur in the postpartum period—defined by the World Health Organization (WHO) as the period beginning 1 h after the delivery of the placenta and continuing until 6 weeks (42 days) after delivery [3]. Although women may die at any time during the postpartum period, research has estimated that maternal mortality is extremely high within the first 2 days of childbirth. In Johannesburg, South Africa, for example, a retrospective study of maternal deaths in health facilities found that, of the 17 maternal deaths that occurred within 42 days of caesarean births, 13 (76%) occurred within the first 2 days of delivery [4]. A study by Barnett et al. [5] found that nearly half (48%) of maternal deaths among Indian women occurred within the intrapartum period and up to 48 h after delivery. These deaths are mainly a result of complications such as postpartum hemorrhage—a leading cause of maternal mortality in developing countries, hypertensive disorders, prolonged or obstructed labor, and puerperal sepsis [6].

Receiving postnatal care particularly within the first 2 days following childbirth—defined here as early postnatal care (EPNC)—is critical to the management of complications and detection of postnatal danger signs, which are necessary for protecting maternal health and averting the majority of postnatal maternal deaths [7–9]. Furthermore, early postnatal care offers an opportunity for women to discuss with providers healthy behaviors such as exclusive breastfeeding, proper nutrition during breastfeeding, and use of effective family planning [10], which are critical to maternal and child survival. For these reasons WHO recommends the first postnatal visit within 24 h of childbirth, and a minimum of three additional postnatal visits timed at 3 days (48–72 h), 7–14 days, and 6 weeks after birth [11]. In many developing countries, however, use of early postnatal care is still at very low levels [12–14].

In Uganda the impact of low coverage of EPNC is reflected as high maternal mortality (336 maternal deaths per 100,000 live births) [15]. Given the urgent need to reduce maternal mortality rate to 320 maternal deaths per 100,000 by 2019/20, as outlined in Uganda’s Health Sector Development Plan 2015/16–2019/20, providing appropriate postnatal care within the first 2 days following childbirth has the potential to dramatically avert maternal deaths through early identification of postnatal danger signs.

However, early postnatal care as a critical aspect of maternal survival has received limited attention compared with pregnancy and skilled birth attendance, and most mothers do not receive postnatal care services from skilled health care providers [16]. In Uganda, for example, almost all women (97%) receive antenatal care (ANC) from a skilled provider at least once during pregnancy, while the coverage of skilled birth attendance and postnatal care within 2 days is lower—at 74 and 54%, respectively. After the second day the percentage of women seeking postnatal care declines significantly [15]. This suggests that the postpartum period is relatively neglected in the continuum of care and hence is a missing link in efforts to achieve safe motherhood. Therefore, the low coverage of postnatal care is a challenge that needs to be addressed.

A few studies have investigated early postnatal care attendance in developing countries such as Bangladesh [17], Nepal [13], Sudan [18], and Zambia [19]. Most of these studies found that delivery at health facilities, skilled birth attendance, proximity to health facilities, having at least a secondary education, and receiving postnatal health education after delivery were associated with use of EPNC. In addition, mothers in urban areas and those in households with middle and rich wealth status were more likely to receive EPNC.

The few studies that have been conducted on postnatal care utilization in Uganda have concentrated on postnatal care beyond 2 days of delivery [20, 21], with no attention to factors influencing early postnatal care attendance—the period when maternal deaths are most common. Other studies on Uganda have focused on postnatal care checkups among newborns [20, 22, 23].

To the best of our knowledge, only one study, by Izudi and Amongin (2015), has examined the factors influencing early postnatal care attendance among mothers in Uganda. Their study, which was conducted among postpartum women, found that only about one-fifth (19%) of the women received a postnatal checkup during the EPNC period. The study also reported that maternal unemployment, lack of information about postnatal schedules, and delivery at public health facilities compared with delivery at private facilities, reduced women’s receipt of early postnatal care (Izudi and Amongin 2015). However, the study had several limitations. It was restricted to only one geographic region, Eastern Uganda, which is not representative of Uganda’s 15 sub-regions, and it focused only on women seeking postnatal care at health facilities. Finally, the sample size comprised only 357 women.

In contrast, the present study is based on the nationally representative 2016 Uganda Demographic and Health Survey (UDHS), which covered the entire country, focused on deliveries at home as well as at health facilities, and comprised a large representative sample of women. Analysis of a national-level study is important to developing strategies to improve EPNC coverage and consequently to reduce Uganda’s high level of maternal mortality.

### Conceptual framework

**Fig. 1:**
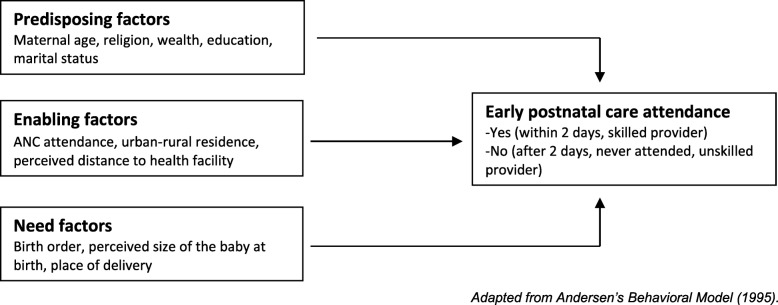
Conceptual framework for determinants of early postnatal care attendance. Adapted from Andersen’s Behavioral Model (1995)

The conceptual framework for this study is shown in Fig. 1. Andersen’s Behavioral Model of health service utilization provided a relevant framework for understanding factors that shape use of early postnatal care [24]. This framework proposes that use of health care services, including postnatal care, is a function of three sets of characteristics—predisposing characteristics, such as age, religion, wealth, and marital status [12, 18, 21, 25, 26]; enabling characteristics, such as ANC attendance and distance to a health facility [21, 27, 28] and need characteristics, such as place of delivery, birth order, and size of the baby at birth [12, 26].

Predisposing factors can influence early postnatal care attendance through several pathways. For example, older maternal age may lead some women to believe that early postnatal care attendance is not critical for optimal health outcomes due to confidence gained from previous pregnancies and births [29]. In contrast, younger women might have better knowledge of maternal health care services due to improvements in educational opportunities for women in recent years, leading to more use of EPNC.

Religion is important in shaping beliefs, norms, and values related to the use of maternal health care services, and may either hinder or facilitate women’s use of EPNC. For example, a study conducted in Ethiopia found that Muslim women were less likely to seek maternal health care compared with other religious groups [30]. Low rates of maternal health service utilization in developing countries have been attributed in part to low levels of involvement in maternal health among Muslim men. Studies by Mosiur Rahman et al. [31] in Bangladesh and Ganle [32] in Ghana found that most Muslim women required permission from their husbands before pursuing activities outside the home, including seeking maternal services at health facilities.

Wealth status has been hypothesized to affect postnatal care attendance, particularly among women who deliver at home and would like to seek postnatal care services immediately after childbirth but lack the resources to do so because these services are often expensive [14, 31]. Employment can increase women’s financial ability to use good-quality medical care and can empower women to take part in the decision-making process about their own health care [33, 34].

Married women may have support from their partners and are therefore more likely to attend EPNC compared with unmarried women [35]. Higher levels of education can enhance female autonomy and help women develop greater confidence and capability to make decisions about their own health, thereby influencing their access to EPNC services [12, 14, 28, 36]. Additionally, education can improve women’s knowledge or awareness of sexual and reproductive health issues. Education coupled with better access to media [37–39] can broaden women’s knowledge of how access to postnatal care can improve their health status and survival.

Enabling factors such as ANC attendance, place of residence, and perception of whether distance to the health facility is a problem could be positively associated with receipt of postnatal care. ANC visits provide an excellent opportunity for providers to deliver adequate counselling regarding the postnatal danger signs and symptoms, enhancing women’s knowledge of possible postpartum complications and the benefits of EPNC services [14, 40]. Hence, women who attend the four or more antenatal care visits recommended by WHO are more likely to use EPNC compared with women who attend fewer than four visits. Place of residence (rural or urban) is another variable that may affect the use of EPNC [28]. Urban residents generally live closer to health care facilities than their rural counterparts. Studies have indicated that geographical distance to a health facility is closely associated with health service utilization [14, 41]. Women who deliver at home may be particularly concerned about the distance to a health facility, thus affecting their use of EPNC.

Further, need factors also influence women’s use of EPNC services. Need factors are known to pose risks to women and newborns and may influence women to seek early postnatal care. For example, women who deliver at a health facility are more likely to receive early postnatal services compared with home deliveries [14, 42]. Also, because of the perceived risk associated with a first pregnancy, women are more likely to seek maternal health care services for first-order births than higher-order births [43]. Having more children may also cause resource constraints, which could have a negative effect on receiving health care [44, 45].

Even though EPNC service utilization plays a critical role in reducing maternal mortality, little is known about its determinants in Uganda. Thus, understanding the factors related to EPNC utilization is critical for countries like Uganda with a high maternal mortality ratio. This study therefore aims to investigate the determinants of early postnatal care attendance among women in Uganda who had a child within 2 years preceding the 2016 UDHS. The study attempts to answer the following research question: What are the determinants of EPNC attendance? We hypothesize that women who deliver at a health facility, women who attend at least four antenatal care visits for their recent birth, and women who perceive that distance from a facility does not hinder their access to health care are more likely to receive EPNC.

## Methods

### Study design and setting

This paper was based on secondary data from the 2016 Uganda Demographic and Health Survey. Authorization to use these data (accessed from The DHS Program website) was obtained upon providing a brief description of our study (The DHS Program 2018). The DHS uses a two-stage cluster sampling design to generate a nationally representative sample of women age 15–49 and men age 15–59 in sampled households. The first stage involves selecting the clusters while the second stage involves selection of households for interview. Stratification of urban and rural areas was taken into account. Details on the sampling procedure are described in the UDHS final report [15]. This analysis used the women’s individual recode data file.

### Study population

**Fig. 2:**
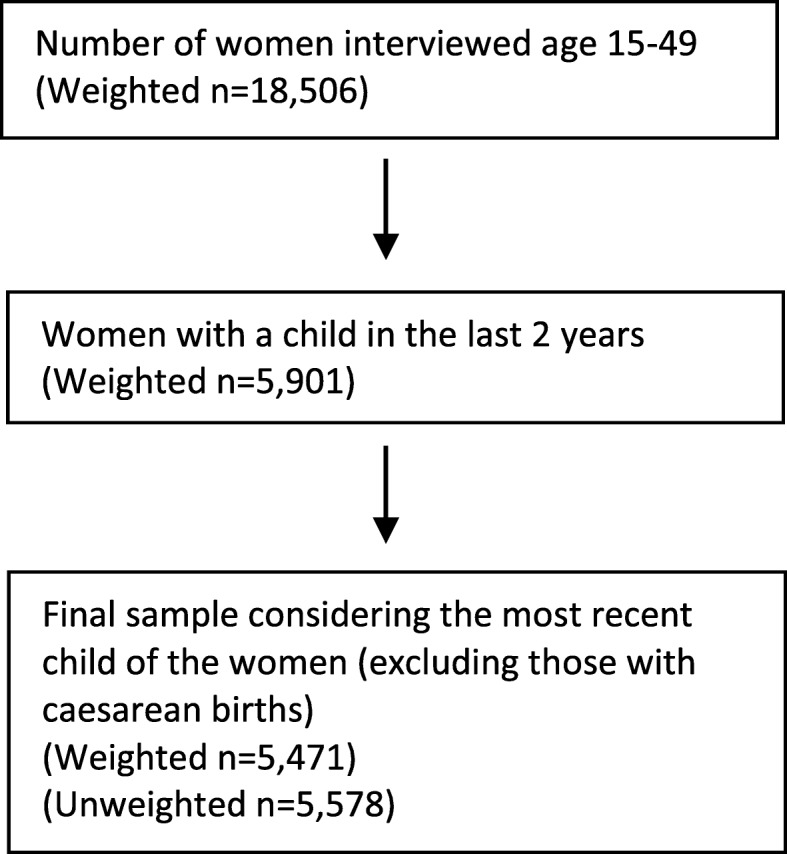
Sample selection criteria

Figure 2 shows the sample selection criteria used for this study. The study sample comprised women who delivered a child in the 2 years preceding the 2016 UDHS and who never had a caesarean birth. Approximately 430 women with caesarean births were excluded from the analysis sample because these births were likely to have received postnatal care regardless of the mother’s characteristics. In our analysis, only the most recent birth in the last 2 years was used to avoid recall bias, especially among women who may have had more than one birth in the last 2 years. In addition, analysis of EPNC use was limited to the mother and not the newborn child. The final sample comprised 5471 women.

### Outcome variable

In the DHS Women’s Questionnaire, all women who had a birth in the 5 years preceding the survey were asked about the checks on their health after delivery while at the health facility or at home. Women were also asked about the timing of their first postnatal checkup after delivery and the provider of the health check. With regard to the provider, this analysis focused on skilled health providers (doctors, nurses, midwives, and medical assistants/clinical officers) because skilled care immediately after delivery is a key strategy in reducing the risk of maternal morbidity and mortality (Bailey et al. 2009).

Therefore, the outcome variable for this study was “early postnatal care attendance,” defined as having received a postnatal checkup from a skilled health provider within 2 days after childbirth. The outcome variable was coded into a binary variable representing having attended postnatal care within 2 days after delivery: Women who had a postnatal checkup by a skilled provider within 2 days of delivery were coded 1. Women who did not attend postnatal care within 2 days of delivery were coded 0, including women who did not receive any postnatal checkup within 2 days, women who never received a checkup at all, and women who were checked within 2 days but by an unskilled provider.

### Independent variables

The independent variables included in this study were mother’s age, residence, education, religion, wealth status, marital status, occupation, antenatal care attendance, place of delivery, perceived size of the child at birth, birth order, women’s perception of whether or not distance to the health facility hinders her access to medical care, and exposure to mass media. Mother’s age was grouped into five categories: 15–19, 20–24, 25–29, 30–34 and 35–49. Age groups 40–44 and 45–49 had only a few cases and were regrouped with age 35–39. Residence was classified as either rural or urban. The education variable was defined as the highest level of education attained by the respondent and was divided into three categories—none, primary, and secondary or higher education. As the large majority (85%) of Uganda’s population belongs to Anglican, Catholic, and Muslim religions (Uganda Bureau of Statistics 2016), we grouped religion into Anglican, Catholic, Muslim, and Others. The category “Others” comprised Pentecostals and smaller religious groups such as Seventh Day Adventists (SDAs) and Orthodox.

Wealth status was categorized in household quintiles—as poorest, poorer, middle, richer, and richest, as per the DHS convention. With regard to marital status, being formerly married (divorced or widowed) and unmarried (both referring to currently unmarried women) did not have enough cases to warrant separate categories as recoded by the DHS, leading us to combine the two groups. Therefore, marital status was collapsed into two groups: unmarried and married. Occupation was measured as the respondent’s occupation and regrouped into three categories: not working, professional employment (a merger of all professional, clerical, sales, and nonprofessional jobs), and agricultural employment.

In the DHS, use of antenatal care was recorded as a continuous variable; however, given that WHO recommends at least four antenatal care visits for pregnant women, ANC attendance was categorized into two categories: four or more visits and fewer than four visits. Place of delivery was regrouped into three categories: home or other location, public health facility, and private health facility. The DHS asked women about their perception of the size of their baby at birth. The DHS groups were: very large, larger than average, average, smaller than average, very small, and don’t know. Since “very large” had few cases, it was grouped with “larger than average.” “Very small” also had few cases so was grouped with “smaller than average.” The category “don’t know” had very few cases so it was recorded as missing. Perception of the size of the baby was therefore regrouped into three categories: large, average, and small. Birth order was collected as a continuous variable and was grouped into four categories: first birth, second or third, fourth or fifth, and sixth or more.

In the DHS, women were asked about their perception of whether or not distance to the health facility hinders access to medical care. Perceived distance to the health facility was grouped into two categories as a variable: distance is a problem, and distance is not a problem. Access to media was defined as whether the woman had heard family planning messages on radio or television, or read a newspaper in the past few months preceding the survey. Access to media was categorized as no or yes.

### Statistical analysis

Three types of statistical analyses were performed. First, descriptive statistics were presented for women’s dependent and independent variables. Second, cross-tabulations with chi square tests were run to determine the association between early postnatal care attendance and the independent variables. A *p*-value of < 0.20 was used to determine the inclusion of independent variables into the multivariate model. Third, logistic regression was used to examine the association between use of early postnatal care and selected independent variables. Results were reported as adjusted odds ratios. The level of statistical significance using *p*-values was set at *p* < 0.05. Important demographic variables based on the literature on early postnatal care were also retained in the logistic regression models, in addition to the variables chosen from the bivariate analysis. Hosmer-Lemeshow goodness-of-fit test was performed to test for the model’s goodness-of fit. Stata version 15 was used to perform all the analyses. All analyses were weighted in order to take into consideration complex survey design, using the *svy* command in Stata.

## Results

Results presented in Table 1 show that out of the weighted sample of 5471 women, 50% received postnatal care in the first 2 days after childbirth. About one-tenth of the sample (12%) comprised women age 15–19, while close to one-third of women (31%) were age 20–24, and about one-quarter (24%) were age 25–29. The mean age was 27 years. The majority of women were rural (80%), had attained primary education (62%), and were married (84%).

**Table 1:** Socio-demographic and maternal characteristics of women (UDHS 2016)

Characteristic	Frequency (***n*** = 5471)	Percent (%)
**PNC utilization**		
Within 2 days	2711	49.5
Not within 2 days	2760	50.5
**Mothers age (mean age) years**	27	
15–19	651	11.9
20–24	1679	30.7
25–29	1327	24.3
30–34	964	17.6
35–49	851	15.6
**Residence**		
Urban	1077	19.7
Rural	4394	80.3
**Highest level of education**		
None	544	9.9
Primary	3393	62.0
Secondary or higher	1534	28.0
**Religion**		
Anglican	1702	31.1
Catholic	2095	38.3
Muslim	803	14.7
Others	871	15.9
**Wealth status**		
Poorest	1283	23.6
Poorer	1202	22.0
Middle	1049	19.2
Richer	967	17.7
Richest	964	17.6
**Marital status**		
Unmarried	855	15.6
Married	4616	84.4
**Occupation**		
Not working	1019	18.6
Professional employment	1954	35.7
Agriculture-related employment	2494	45.6
**ANC attendance**		
4 or more visits	3257	59.5
Less than 4 visits	2214	40.5
**Place of delivery**		
Home/other	1390	25.4
Public facility	3222	58.9
Private facility	860	15.7
**Size of baby**		
Large	1391	25.9
Average	2840	52.9
Small	1140	21.2
**Birth order**		
First	1183	21.6
Second or third	1880	34.4
Fourth or fifth	1209	22.1
Sixth or more	1200	21.9
**Perceived difficulty of distance to health facility**		
Problem	2280	41.7
Not a problem	3192	58.3
**Access to media messages**		
No	1810	33.1
Yes	3661	66.9

Table 1 also shows that about two-fifths of women (41%) did not attend the recommended four antenatal care visits for their most recent pregnancy. The majority of women delivered their last child at a public health facility (59%), while 16% delivered at a private facility, and 25% delivered at home. For most women, their most recent birth was their second or third child. More than half of the women (53%) said their most recent child was of average size at birth. Two-thirds of the women (67%) had been exposed to family planning messages via radio, television, or newspapers.

### Bivariate analysis results of early postnatal care utilization

Table 2 shows the percentage of women who received postnatal care in the first 2 days (EPNC) according to socio-demographic and maternal characteristics. The findings show that residence, education level, religion, wealth status, marital status, occupation, ANC attendance, place of delivery, birth order, perceived accessibility of health facilities, and access to mass media messages were associated with greater use of EPNC. Women in urban areas were more likely to use EPNC than women in rural areas, at 63% versus 46%. Nearly two-thirds of the women with secondary education or higher (64%) received EPNC compared with less than half of women with no education (47%) and women with a primary education (44%). Among Muslim women, 58% received EPNC compared with 47% of Anglicans, 49% of Catholics, and 49% of other religious denominations.

**Table 2:** Percentage distribution of women by use of early postnatal care (UDHS 2016)

Characteristic	Frequency (***n*** = 5471)	Attended PNC within 2 days (***n*** = 2711)	***p***-value
**Mother’s age**			
15–19	651	328 (50.4)	0.202
20–24	1679	834 (49.7)	
25–29	1327	672 (50.7)	
30–34	964	494 (51.2)	
35–49	851	382 (44.9)	
**Residence**			
Urban	1077	676 (62.7)	< 0.001
Rural	4394	2035 (46.3)	
**Highest level of education**			
None	544	254 (46.8)	< 0.001
Primary	3393	1481 (43.7)	
Secondary/higher	1534	976 (63.6)	
**Religion**			
Anglican	1702	801 (47.0)	0.002
Catholic	2095	1019 (48.6)	
Muslim	803	465 (57.9)	
Others	871	427 (49.0)	
**Wealth status**			
Poorest	1283	589 (45.7)	< 0.001
Poorer	1202	523 (43.5)	
Middle	1049	469 (44.7)	
Richer	967	482 (49.9)	
Richest	964	648 (67.2)	
**Marital status**			
Unmarried	855	453 (52.9)	0.082
Married	4616	2258 (48.9)	
**Occupation**			
Not Working	1019	483 (47.5)	< 0.001
Professional employment	1954	1119 (57.3)	
Agricultural employment	2494	1106 (44.4)	
**ANC attendance**			
4 or more visits	3257	1768 (54.3)	< 0.001
Less than 4 visits	2214	943 (42.6)	
**Place of delivery**			
Home/other	1390	127 (9.1)	< 0.001
Public facility	3222	2024 (62.8)	
Private facility	860	561 (65.2)	
**Size of baby**			
Large	1391	714 (51.4)	0.252
Average	2840	1429 (50.3)	
Small	1140	543 (47.6)	
**Birth order**			
First	1183	675 (57.1)	< 0.001
Second or third	1880	954 (50.8)	
Fourth or fifth	1209	569 (47.1)	
Sixth or more	1200	513 (42.8)	
**Perceived difficulty of distance to health facility**			
Problem	2280	976 (42.8)	< 0.001
Not a problem	3192	1735 (54.4)	
**Access to media messages**			
No	1810	746 (41.2)	< 0.001
Yes	3661	1965 (53.7)	

Receipt of EPNC was more common among women who attended at least four ANC visits for their most recent birth, at 54%, compared with women who did not receive the recommended four or more ANC visits, at 43%. The percentage of women receiving EPNC was higher among women who delivered at a health facility, either a public facility (63%) or private facility (65%), compared with women who delivered at home (9%). Receipt of EPNC varied with women’s access to mass media. A higher proportion of women who had access to media (54%) received EPNC compared with those who had no access to media (41%). Maternal age and perceived size of the baby at birth were not significantly associated with use of EPNC.

**Fig. 3:**
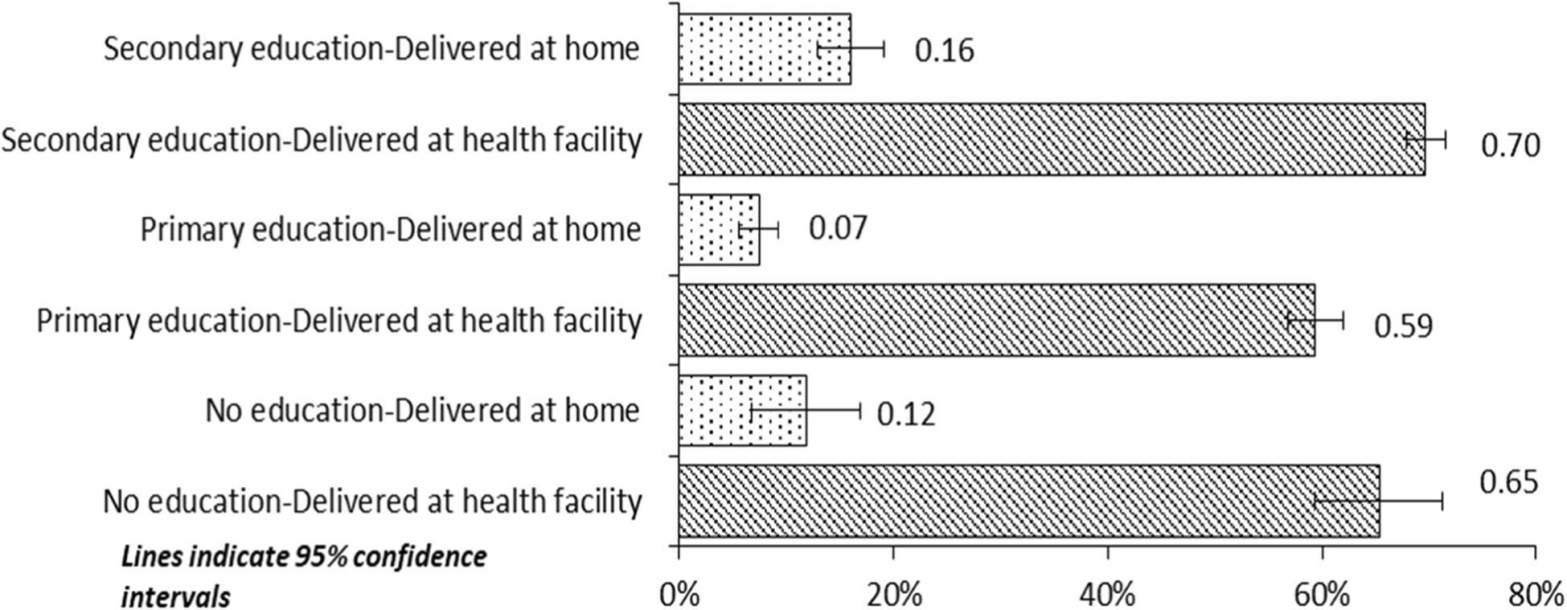
Receipt of EPNC, by women’s education and place of delivery (health facility or home)

Figure 3 illustrates EPNC use by women’s level of education and place of delivery. Overall, receipt of EPNC was much higher among women who delivered at a health facility compared with delivery at home, irrespective of education status. This suggests that delivery at a health facility is an important determinant of EPNC attendance. Furthermore, as Fig. 3 shows, among mothers who delivered at a health facility, the proportion with no education was comparable to the proportion with secondary or higher education (65% versus 70%). Regardless of the place of delivery, the proportion of mothers with secondary education receiving EPNC was significantly higher than among those with only a primary education.

### Multivariate analysis

Table 3 shows the results of the multivariate logistic regression of EPNC attendance. Although the bivariate analysis indicated that women’s age was not significantly associated with early postnatal care utilization, this variable was considered for inclusion in the logistic regression model based on previous literature. However, the variable was omitted because of multicollinearity—that is, women’s age was strongly correlated with birth order (correlation coefficient (r) =0.80). Owing to the greater importance of birth order as a key factor influencing early postnatal care attendance (Khanal et al. 2014), birth order was included in the logistic regression model while age was dropped.

**Table 3:** Multivariate analysis of the factors associated with early postnatal care attendance among women age 15–49 with a birth in the 2 years preceding the survey (UDHS, 2016)

Characteristic	Frequency (***n*** = 5471)	aOR	95% CI
**Residence**			
Urban	1077	1.16	0.89–1.51
Rural	4394	1.00	
**Highest level of education**			
None	544	1.33*	1.04–1.69
Primary	3393	1.00	
Secondary/higher	1534	1.45**	1.22–1.74
**Religion**			
Anglican	1702	0.95	0.79–1.95
Catholic	2095	1.00	
Muslim	803	1.17	0.92–1.47
Others	871	1.04	0.84–1.30
**Wealth status**			
Poorest	1283	1.00	
Poorer	1202	0.76*	0.59–0.97
Middle	1049	0.69*	0.53–0.91
Richer	967	0.66*	0.50–0.86
Richest	964	0.95	0.67–1.35
**Marital status**			
Unmarried	855	1.12	0.91–1.38
Married	4616	1.00	
**Occupation**			
Not working	1019	0.72*	0.58–0.91
Professional employment	1954	1.03	0.87–1.21
Agricultural employment	2494	1.00	
**ANC attendance**			
4 or more visits	3257	1.20*	1.04–1.39
Less than 4 visits	2214	1.00	
**Place of delivery**			
Home/other	1390	1.00	
Public facility	3222	15.28**	11.92–19.58
Private facility	860	15.68**	11.89–20.67
**Birth order**			
First	1183	1.00	
Second or third	1880	0.87	0.72–1.05
Fourth or fifth	1209	0.98	0.77–1.24
Sixth or more	1200	1.00	0.79–1.28
**Perceived difficulty of distance to health facility**			
Problem	2280	1.00	
Not a problem	3192	1.20*	1.03–1.39
**Access to media messages**			
No	1810	1.00	
Yes	3661	1.31**	1.13–1.52

*p < 0.05, **p < 0.01, ***p < 0.001; *aOR* adjusted odds ratio, *CI* Confidence Interval

Multivariate logistic regression analysis identified the following statistically significant correlates of EPNC attendance: women’s level of education, household wealth status, employment status, receipt of four or more ANC visits, place of delivery, perception of the accessibility of a health facility, and access to mass media. Women with no formal education (aOR = 1.33, CI 1.04–1.69) and those with a secondary or higher education (aOR = 1.45, CI 1.22–1.74) were more likely to use early postnatal care compared with women with a primary education. Women in households in the poorer (aOR = 0.76, CI 0.59–0.97), middle (aOR = 0.69, CI 0.53–0.91), and richer (aOR = 0.66, CI 0.50–0.86) wealth quintiles were less likely to attend EPNC compared with women in the poorest wealth quintile. Unemployed women had lower odds (aOR = 0.72, CI 0.58–0.91) of attending postnatal care within 2 days after childbirth compared with women employed in the agricultural sector.

Also, women who attended at least four ANC visits for their recent pregnancy had higher odds of attending postnatal care within 2 days after childbirth (aOR = 1.20, CI 1.04–1.39) compared with those who attended fewer than four ANC visits. Women who delivered at a health facility—either a public hospital (aOR = 15.28, CI 11.92–19.58) or a private facility (aOR = 15.68, CI 11.89–20.67)—were more likely to attend EPNC compared with those who delivered at home. Women who perceived that distance to the health facility did not hinder their access to health care were more likely (aOR = 1.20, CI 1.03–1.39) to attend EPNC than those who perceived distance to the facility as a problem. Likewise, the odds of attending EPNC were higher among women who were exposed to mass media messages (aOR = 1.31, CI 1.13–1.52) compared with women who never accessed mass media.

Place of residence, religion, marital status, and birth order were not significantly associated with EPNC attendance.

## Discussion

Maternal mortality remains a challenge in achieving the Sustainable Development Goal target of reducing the global maternal mortality ratio to fewer than 70 maternal deaths per 100,000 live births in low-resource settings like Uganda. Prior studies have noted the importance of early postnatal checkups in improving maternal survival [18, 19, 46, 47]. This study investigated the determinants of early postnatal care attendance (receipt of postnatal care within 2 days after childbirth) among women in Uganda based on data from the 2016 UDHS. Our study revealed that one-half of women received early postnatal care. This result is much higher than the levels reported in studies among postpartum women in Nepal [48], Nigeria [14], and Ethiopia [12], but lower than the 68% reported in Benin [8] and 63% in Zambia [19].

Low levels of postnatal care attendance in Uganda may be explained by the fact that a quarter of deliveries occur at home [15]. Most of these home deliveries occur among women who reside in rural areas, women in the poorest household wealth quintile, and those who perceive that distance to a health facility hinders their access to medical care (results from preliminary analysis, data not shown). It is also possible that women who deliver at home might not perceive themselves as needing a medical checkup; having survived delivery, the woman might then be considered “out of danger.” Perhaps only women who develop observable complications would seek postnatal care services within 2 days of childbirth.

Another possible explanation for low levels of EPNC, despite the relatively high proportion of facility-based deliveries, is that women who deliver at a health facility do not stay in the hospital for long after the delivery. They might have been discharged early (< 24 h for vaginal deliveries) [11], before receiving their first postnatal check [49]. It also seems possible that the way the DHS survey question is phrased, “…*Did anyone check on your health?*” may not be clear enough for many women to understand [50]. Our results emphasize the need to strengthen attendance at EPNC by recognizing and addressing barriers to its use.

Results of the multivariate analysis indicated the following factors as correlates of EPNC attendance in Uganda: woman’s education level, household wealth status, employment status, ANC attendance, place of delivery, whether distance to the health facility is perceived as a problem, and access to media. Women with secondary or higher education are more likely to receive EPNC compared with women with primary education. This result is in accord with recent studies showing that higher education was significantly associated with postnatal care attendance among women [12–14]. A possible explanation for this might be that education is associated with increased awareness of basic health services and with being informed about health risks, which can lead to improved health-seeking behavior. Having a secondary or higher education may also give women a sense of empowerment to voice their opinions in decisions having to do with their own health.

Our results also indicated that uneducated women were more likely to attend early postnatal care than those with primary education. This finding is not consistent with those of other studies conducted in Africa [14, 51, 52] and other developing countries [48, 53], which confirm that better educated women are more likely to seek postnatal care services. This counterintuitive finding may be partially explained by the notion that uneducated mothers may lack confidence in their own knowledge of health and are therefore more compliant with health professionals’ recommendations.

The results also showed that the likelihood of EPNC attendance was lower among unemployed women compared with women employed in the agricultural sector. This could be attributed to many factors, including the fact that unemployed women are more likely to be economically dependent and consequently unable to access and use maternal health services recommended by health workers [33, 34].

In this study, use of EPNC services was found to be higher among mothers who had access to mass media. This is consistent with results from studies in Nigeria [54] and Malawi [37]. Besides offering entertainment, mass media informs and educates, increasing women’s access to knowledge and improving their ability to seek health care.

Among the enabling factors, ANC attendance and perceptions of whether distance to a health facility is a problem were significantly associated with attending EPNC. Receipt of the recommended minimum of four ANC visits also emerged as a statistically significant correlate of early postnatal care utilization. Our study found that mothers who attended four or more antenatal care visits, as recommended by WHO, had higher odds of receiving a postnatal checkup compared with women who did not attend at least four ANC visits. These results support previous research linking use of antenatal care to use of postnatal care [31, 48]. A possible explanation for this result is that antenatal care visits expose pregnant women to counselling and education about their own health and the care of their children. This exposure may be particularly advantageous in low-resource settings like Uganda, where health-seeking behaviors are inadequate, access to health services is limited, and most mothers are rural, poor, and with little formal education.

Likewise, our finding that distance to a health facility is not a problem in accessing health care was a statistically significant correlate of EPNC has also been reported elsewhere in sub-Saharan Africa [41, 55]. Long distances limit the willingness and ability of postpartum women to seek postnatal care due to the physical difficulties of travel and high costs of motorized transport [18]. In addition, multiple demands including caring for the newborn and older children often prohibit women from seeking postnatal care where proximity to a health facility is perceived to be a challenge.

It is also possible that women who deliver at home are limited by cultural norms that may prohibit them from leaving the house for a few days after the child is born, as found in Nepal [56] and India [57]. In rural Uganda traditional beliefs have prevented postpartum mothers with mental illness from seeking timely care, as they were made to believe that the illness was due to witchcraft [58].

With regard to need factors, mothers who delivered at a health facility (either public or private) were 15 times more likely to receive a postnatal care checkup within 2 days of childbirth than women who delivered at home—a finding that emphasizes the importance of health facility delivery. This finding is in agreement with the study by Dahiru and Oche [28] of the determinants of utilization of maternal and child health services, including postnatal care, using data from the Nigeria DHS, and with the study in Zambia by Chungu et al. [19]. Delivering at a health facility increases the chances of skilled birth attendance, which plays a vital role in ensuring that mothers receive comprehensive care, including postnatal care soon after delivery. Another possible explanation for this finding is that delivery at a health facility ensures access to emergency obstetric care to deal with complications during labor, which may also expose women to postnatal care.

However, our results also indicated that 37% of women who delivered at a public health facility and 35% who delivered at a private health facility did not receive any postnatal care within 2 days of childbirth. This finding is in agreement with the study in Zambia by Chungu et al. [19] reporting that 20% of women who delivered at a health facility did not attend postnatal care within 48 h of delivery. This finding is counterintuitive because one would expect that all health facility deliveries with skilled health providers would receive EPNC. A plausible explanation for this finding is early discharge from the facility too soon after delivery to allow for EPNC. Another explanation could be limited staffing at health facilities and inadequate training of health staff, which constrain provision of EPNC services, as has been reported in a study in the Katakwi and Mubende districts of Uganda that documented challenges in providing integrated health services, including postnatal care [59]. Missing the opportunity to provide essential postnatal care for facility deliveries unnecessarily puts the lives of mothers at risk.

### Limitations of the study

One major limitation of using DHS data is it does not address any elements related to the quality of care received. The postnatal care module in the survey did not capture information about clinical screenings that were performed or assessments that were done (eg questions such as, “Was your temperature taken during the postnatal visit?”). Another limitation is the cross-sectional nature of the DHS survey, which means that causality cannot be established between the variables, but only associations. The study is further limited by the fact that it is based on retrospective information provided by the survey respondents, which may be subject to recall bias. However, such bias was minimized to some extent by restricting the study to mothers with a birth within 2 years of the survey. In addition, all study results relate to the most recent birth.

## Conclusions

This study has revealed that half of Ugandan women attended postnatal care services within 2 days of childbirth. Factors that were positively associated with use of EPNC included: secondary or higher education, having four or more recommended ANC visits during pregnancy, delivery at a health facility (public or private), perception that distance to the health facility does not hinder access to health care, and access to media.

The results underscore the importance of supporting facility-based deliveries and also ramping up efforts for home visits offering EPNC. This calls for: first, that the Government of Uganda and nongovernmental organizations should place more emphasis on EPNC training of health workers, such that mothers are counselled during ANC sessions about the life-saving potential of early postnatal care and that EPNC is provided before hospital discharge; second, that interventions to increase the use of EPNC should target women with primary education; third, because a quarter of births occur at home (Uganda Bureau of Statistics and ICF 2018), the current capacity of health facilities should be strengthened through community-based promotion programs involving home visits by midwives and community health workers and providing maternal health services within a reasonable distance to households; and fourth, that maternal health stakeholders and the Government of Uganda should utilize mass media such as television and radio to sensitize women about the availability and importance of EPNC for maternal survival. Further research is required to establish the factors that contribute to nonuse of EPNC for women who deliver at a health facility.

## Data Availability

The dataset used and /or analyzed during the current study is available at: https://dhsprogram.com/customcf/legacy/data/download_dataset.cfm?Filename=UGIR7ADT.zip&Tp=1&Ctry_Code=UG&surv_id=504&dm=1&dmode=nm. Registration is required. We used the UGIR70FL (Individual Recode –Women with completed interviews – Uganda, 2016).

## Abbreviations

ANC: Antenatal care
EPNC: Early postnatal care
UDHS: Uganda Demographic and Health Survey
USAID: United States Agency for International Development
WHO: World Health Organization
